# Valorizations of Sweet Cherries Skins Phytochemicals by Extraction, Microencapsulation and Development of Value-Added Food Products

**DOI:** 10.3390/foods8060188

**Published:** 2019-06-01

**Authors:** Adelina Ștefania Milea, Aida Mihaela Vasile, Adrian Cîrciumaru, Loredana Dumitrașcu, Vasilica Barbu, Gabriela Râpeanu, Gabriela Elena Bahrim, Nicoleta Stănciuc

**Affiliations:** 1 Dunărea de Jos University of Galati, Faculty of Food Science and Engineering, Domnească Street 111, 800201 Galati, Romania; adelina.milea@ugal.ro (A.Ș.M.); aida.vasile@ugal.ro (A.M.V.); loredana.dumitrascu@ugal.ro (L.D.); vasilica.barbu@ugal.ro (V.B.); grapeanu@ugal.ro (G.R.); gbahrim@ugal.ro (G.E.B.); 2 Dunărea de Jos University of Galati, Cross-Border Faculty of Humanities, Economics and Engineering, Domnească Street 47, 800201 Galati, Romania, Romania; adrian.circiumaru@ugal.ro

**Keywords:** sweet cherry, anthocyanins, microencapsulation, whey proteins, value-added food products

## Abstract

Sweet cherries are processed in various ways, leading to significant amounts of underutilized by-products that can potentially be used as a source of bioactive compounds, including antioxidants. The present study focuses on identifying ways to exploit bioactive compounds from sweet cherry skins, namely the extraction, microencapsulation, and functionalizing of some food product to obtain added value. The anthocyanins from skins were extracted and encapsulated in a combination of whey proteins isolate and chitosan by freeze-drying, with an encapsulation efficiency of 77.68 ± 2.57%. The powder showed a satisfactory content in polyphenols, of which anthocyanins content was 14.48 ± 1.17 mg cyanidin 3-glucoside/100 g dry weight (D.W.) and antioxidant activity of 85.37 ± 1.18 µM Trolox/100 g D.W. The powder was morphologically analyzed, revealing the presence of coacervates, ranging in size from 12–54 μm, forming large spheresomes (up to 200 μm). The powder was used as a functional ingredient to develop two value-added food products, namely yoghurt and marshmallows. The powder was tested for its prebiotic effect on *L. casei* 431^®^ in the yoghurt samples during 21 days at 4 °C, when a decrease in viability was found, up to 6 log CFU·g^−1^. The anthocyanins and antioxidant activity decreased in yoghurt and increased in marshmallows during storage time. The obtained results support the potential use of extracts from underutilized sources in the development of functional ingredients and value-added food products.

## 1. Introduction

The processing of fruits and vegetables in order to extract oils, juices and sugars results in significant amount of skins, stems and seeds. Usually, these by-products are disposed of in the environment, which raises a number of serious problems due to their poor biological stability, significant nutritional value, high concentration of organic compounds, high water activity, poor oxidative stability and optimum enzymatic activity [1]. The use of these by-products can bring economic and environmental benefits as they are considered of no commercial value and rich in compounds with a potentially positive effect for human health. Therefore, food industries are constantly trying to utilize the resultant by-products, which may be considered as highly nutritional and functional food ingredients, such as polysaccharide, vitamins, minerals, dietary fiber and biologically active compounds [1]. Selected by-products are a significant source of natural antioxidants and antimicrobials, being considered an excellent way to improve food quality and shelf-life without introducing undesirable chemical preservatives [2].

Sweet cherries (*Prunus avium*) are consumed in large quantities due to their attractive colour, sweetness and wealth of antioxidants and nutrients. The fruits contain phenolic compounds, in which 60–74% of the phenols consist mainly of hydroxycinnamates [3,4], anthocyanins, flavan-3-ols [5]. It has been reported that anthocyanins are responsible for the attractive colour of cherries, with quantities ranging from a few mg/100 g to about 700 mg/100 g in dark cherries [6]. Wani et al., [7] suggested that the predominant anthocyanins present in dark-coloured cherries are cyanidin 3-rutinoside and cyanidin 3-glucoside, while hydroxycinnamates, neochlorogenic acid and p-coumaryl quinic acid have been found in adequate quantities [8]. The health benefits of cherries are related to the strong antioxidant capacity of the sweet cherries and were described by Wani et al., [7] as: supporting weight loss [8], neuroprotective effects [9], cancer preventive properties [10], pain relief from inflammation and arthritis [11], exercise induced muscle damage symptoms [12], prevention of oxidative stress [13] and protection against neurodegenerative diseases [4].

Due to their short shelf life (7–10 days), sweet cherries are processed into a variety of food products such as: marmalades, alcoholic beverages, jams, jelly fruits, and juices. The large volume of processed cherries results in significant quantities of by-products, including skins. However, few studies can be found in the literature concerning the reuse of skins.

Sweet cherries skins are a rich source of anthocyanins, being able to increase the life quality through its properties. Anthocyanins are natural pigments, having a large number of biological activities, including: antioxidant, anti-inflammatory, anticancer, antimutagenic, chemo-preventive and inhibiting α-glucosidase activity, UV protection and pathogens [14]. Despite all those benefits of anthocyanins, there is a real concern regarding their phenolic structure. They are easy degradable because of their sensitivity to many factors such as pH, temperature, light, oxygen. Also, they undergo degradation during digestion, resulting in low absorption and bioavailability [15]. Therefore, a method involving their protection and the controlled release of bioactive compounds could be the key to encapsulation in some complex matrices. It is known that whey protein (WP) and chitosan have already been used in many studies for the encapsulation of bioactive compounds. Whey protein isolate (WPI) from the dairy industry is an important source of β-lactoglobulin, whereas chitosan is the second most abundant polysaccharide in the nature.

Considering the health benefits of anthocyanins and in our continued quest to find new edible sources with functional properties, the aim of this study was to exploit the phytochemicals from sweet cherries skins through extraction and microencapsulation in whey proteins isolates and chitosan by freeze-drying, in order to obtain functional, food-grade ingredients. The obtained extract and powders were characterized in terms of phytochemicals, antioxidant activity and encapsulation efficiency. The microstructure of the particles was analyzed by confocal scanning laser and scanning electron microscopy. Due to its functional properties, especially antioxidant activity, the resulting powders were used to develop two value-added foods, such as yoghurt and marshmallow. The value-added products have been tested for the lactic bacteria viability, as well as the stability of biologically active compounds and antioxidant activity during storage. The obtained results support the potential use of extracts from underutilized sources in the development of functional ingredients and value-added food products.

## 2. Materials and Methods

### 2.1. Chemicals

Whey proteins isolate (protein content 95%) (WPI) was purchased from Fonterra (Auckland City, New Zealand). 2,2-Diphenyl-1-picrylhydrazyl (DPPH), 6-Hydroxy-2,5,7,8-tetramethylchromane-2-carboxylic acid (Trolox), chitosan, ethanol and methanol (HPLC grade), sodium hydroxide, Folin-Ciocalteu reagent and gallic acid were obtained from Sigma Aldrich Steinheim (Darmstadt, Germany).

### 2.2. Sweet Cherries Fruits and Anthocyanins Extraction

Sweet cherries (*Prunus avium* L.) were purchased from the local market (Galați) in June–July 2017. Fruits samples were washed and the skins and seeds were manually separated from the pulp. The skins were washed with distilled water and then blotted on paper towels to remove any residual pulp. Skins were freeze-dried and stored at a temperature of −20 °C until analyses.

The extraction of anthocyanins from freeze-dried sweet cherry skins was performed according to a modified procedure [16], by using the ethanolic method combined with ultrasound assisted extraction. In brief, 10 g of freeze-dried skins powder was grounded and extracted in 80 mL of 70% ethanol solution. The solution was introduced in an ultrasonic bath equipped with a digital control system of sonication time, temperature and frequency (MRC Scientific Instruments, Holon, Israel). The extraction was performed at a constant frequency of 40 kHz, with a constant power of 100 W. Cold water was added to maintain a constant temperature of 40 °C (±0.5 °C). The ultrasound assisted extraction was performed for 30 min, followed by centrifugation at 9000 rpm for 10 min at 4 °C. The supernatant was collected, and the extraction was repeated four times. The collected supernatants were concentrated until dry (RVC 2-18, Christ, Osterode, Germany). Prior to phytochemical characterization and microencapsulation, the extract was dissolved by adding 10 mL of Milli Q water to 1 g of extract.

The resulted extract was characterized in terms of total monomeric anthocyanins content (TAC) (mg cyanidin 3-glucoside (C3G)/g D.W.), total polyphenolic content (TPC, mg gallic acid equivalents (GAE)/g D.W.)), total flavonoids content (TFC, mg catechin equivalents (CE)/g D.W.) and antioxidant activity (mmol Trolox/g D.W.), as previously described in our work [16].

### 2.3. Microencapsulation of Anthocyanins

The microencapsulation of anthocyanins from the sweet cherry skins extract by freeze-drying was performed as described by Oancea et al. [17] with slight changes. Individually, 2 g of WPI and 1 g of chitosan were dissolved in 100 ml of ultrapure water and allowed to stand overnight under stirring at room temperature to allow complete hydration. The two solutions were mixed and the pH was adjusted at 7.0 with 1 M NaOH. After complete hydration, 10 mL of the extract solution was added and stirred for 2 hours at 500 rpm at room temperature. The microencapsulation experiments were performed in duplicates.

### 2.4. Powder Characterization

The methods previously described by Oancea et al. [18] were used in this study to characterized the powder, in terms of encapsulation efficiency, phytochemicals content, and antioxidant activity. Briefly, a colorimetric method, based on the aluminium chloride capacity of forming stable acid complexes with the flavonols was used to determine TFC (expressed as mg CE/100 g D.W.). The Folin-Ciocalteu method was used to determine the content of TPC (expressed as mg GAE/100 g D.W.). For the determination of antioxidant activity, the protocol for measuring antiradical activity on DPPH (2,2-diphenyl-1-picrylhydrazyl) was used and expressed as mmol Trolox/100 g D.W.

For the microencapsulation efficiency evaluation, the procedure described by Saénz et al., [19] was used, as the difference between the anthocyanins retention (AR) and the anthocyanins located in the microcapsule surface (AS). To quantify the AS, 200 mg of powder was mixed with 1 mL of ethanol and methanol (1:1). These dispersions were stirred at room temperature for 1 min and then centrifuged (4000× *g*, 10 min). For AR, 200 mg of powder were accurately weighed and dispersed in 1 mL ethanol, acetic acid, and water (50:8:42). This dispersion was agitated using a Vortex (1 min) and then an ultrasonicator twice for 30 min. The supernatant was centrifuged at 20,000× *g* for 10 min and then filtered. TAC was quantified in supernatants by pH-differential method and expressed as mg C3G/100 g DW.

The microencapsulation efficiency (EE, %) was calculated with Equation (1):

(1) $$EE\%=\frac{\left(AR-AS\right)}{AR}\times 100,$$

### 2.5. Confocal Laser Scanning Microscopy

Confocal analysis focused on the microstructural aspects of the powder obtained by freeze-drying microencapsulation process of sweet cherries skins anthocyanins into WPI and chitosan. The confocal equipment that was used for the study is a Zeiss Axio Observer Z1 inverted microscope model (LSM 710) equipped with a laser scanning system: diode laser (405 nm), Ar laser (458 nm, 488 nm and 514 nm), DPSS (561 nm pumped solid-state diodes), and He Ne-laser (633 nm) (Carl Zeiss MicroImaging, GmbH, Jena, Germany). The powder was stained with two dyes, 4′,6-diamidino-2-phenylindole (DAPI) (1 μg/mL) and Congo Red (40 μM), in a ratio 3:1:1, and observed using a 40× apochromatic objective (numerical aperture 1.4) and the FS49, FS38, FS15 filters. The strong anthocyanin absorption in the visible range was assessed near 550 nm and the reflectance in the green region of the spectrum proved to be sensitive in regards to the anthocyanin content [20]. The excitation wavelengths for DAPI and Congo Red were 358 and 497 nm whereas the emission wavelengths were 463 and 614 nm, respectively. The 3D images were rendered and analyzed with ZEN 2012 SP1 software (Black Edition).

### 2.6. Scanning Electron Microscopy

The powder morphologies were analyzed by scanning electron microscope. Samples were placed on carbon foil and coated with 5 nm gold thickness in Ar atmosphere. Surface micrographs of samples were obtained using the FEI Quanta 200 (SPI Spattering, West Chester, PA, USA) by exposition to accelerated electron beams of 20 kV, pressure of 0.6 mmHg and spot size of 4. The images of the microcapsules were taken at magnifications of 20,000× and 500,000×.

### 2.7. Formulation of Value-Added Food Products

The effect of microencapsulated anthocyanins on *Lactobacillus casei* 431^®^ was tested as described by Oancea et al. [17]. First, *L. casei* 431^®^ lyophilized starter culture was reactivated in UHT whole milk (3.5% fat, Müller, Romania) and incubated under aerobic conditions for 20 minutes at 37 °C, whereas the powder was sterilized under UV for 30 min. Three yoghurt samples were tested by varying the added powder content, namely: **A**—milk with *L. casei* 431^®^ (control sample), **B**—A sample + with 5% of powder and **C**—A sample + with 10% addition of powder. The cell viability of the *L. casei* 431^®^ were tested as described by Oancea et al., [17] at 0, 7, 14 and 21 days of storage at 4 °C using Koch’s serial dilution method. The results were expressed as log CFU·g^−1^ (colony forming units per gram of product) [21].

The functionality of the powder was also tested by incorporation in the marshmallows in a ratio of 5 (S1) and 10% (S2). The products were obtained by whipping egg whites with white sugar, incorporating the lyophilized powder, followed by whipping, pouring and drying at 85 °C for one hour. A control sample without powder addition was also obtained.

### 2.8. Phytochemical Analysis of Value-Added Food Products

The value-added products were tested for TAC and antioxidant activity. An extraction of 1 g of product was performed with 10 mL of 70% ethanol and 1 mL of HCl, followed by centrifugation at 9000× *g* for 20 min at 4 °C and filtration through 0.22 μm-sized Millipore membranes. The TAC and antioxidant activity were determined in the resulting supernatant according to Oancea et al. [17], using differential pH method and DPPH method, and expressed as mg C3G/100 g D.W. and mmol Trolox/100 D.W., respectively.

Since no preservatives were used in the formulation, it was decided to perform a storage stability test. Therefore, the yoghurt sample were stored at 4 °C for 21 days, whereas the marshmallows were packed in plastic bags and stored for seven days at room temperature. For determination of TAC and antioxidant activity, vials were removed every seven days for yoghurt and three days for marshmallows.

### 2.9. Statistical Analysis of Data

Unless otherwise stated, the results are expressed as mean of triplicate samples ± standard deviation. To assess the influence of storage time and sample type on the viability of *Lactobacillus casei* 431^®^, anthocyanins content and antioxidant activity, one-way ANOVA available on Minitab 18 statistical software was used only if normality (Shapiro-Wilk test) and equality of variances test were first passed. If the *p* value resulted in an ANOVA lower than 0.05, the Tukey method was further used to assess the differences between samples.

## 3. Results and Discussion

### 3.1. Sweet Cherries Extract Characterization

The extract showed a TAC of 12.2 ± 0.5 mg C3G/g D.W., whereas TPC, TFC and antioxidant activity were 445.93 ± 5.11 mg GAE/g DW, 152.22 ± 32.78 mg CE/g DW and 10.60 ± 0.44 mmol Trolox/g D.W., respectively.

As regarding the individual anthocyanins content, Turturică et al. [22] suggested that sweet cherries skins extract contains five anthocyanins, as follows: cyanidin 3-rutinoside, cyanidin 3-glucoside, peonidin 3-rutinoside, peonidin 3-glucoside and pelargonidin 3-rutinoside. The major anthocyanin was cyanidin 3-rutinoside (109.03 ± 8.8 mg/100 g D.W.), followed by cyanidin 3-glucoside (83.75 ± 2.9 mg/100 g D.W.), pelargonidin-3-rutinoside (32.99 ± 1.6 mg/100 g D.W.), peonidin 3-rutinoside (8.41 ± 0.9 mg/100 g D.W.) and peonidin 3-glucoside (4.97 ± 0.6 mg/100 g D.W.). Kim et al. [8], suggested also the presence of two anthocyanins in dark-coloured cherries, namely cyanidin 3-rutinoside and cyanidine 3-glucoside.

### 3.2. Encapsulation Efficiency

The encapsulation efficiency of anthocyanins from sweet cherries skins in WPI and chitosan was 77.68 ± 2.57%. Oancea et al. [17] reported a value of 70.28 ± 2.17% for anthocyanins extracted from sour cherries skins and microencapsulated in WPI and acacia gum, whereas when using single protein as a wall material, the encapsulation efficiency ranged from 44.79 ± 0.90% to 64.69 ± 0.24% [11]. When encapsulating anthocyanins from grape skins extract in WPI, Stănciuc et al. [23], suggested an encapsulation efficiency of 94% in case of acacia gum and 99% when using pectin.

Li et al. [24], microencapsulated polyphenolics from ethanolic plum extract by spray drying and reported encapsulation efficiency varying from 76.4 ± 1.9% and 87.4 ± 1.4% as a function of air inlet temperature, core content and feed solids concentration. Akhavan Mahdavi et al. [25], suggested that microencapsulation efficiency depends on type of wall material and core/wall ratio. These authors used maltodextrin, gum Arabic and gelatin to microencapsulated anthocyanins from barberry (*Berberis vulgaris*) extract by spray drying, reporting encapsulation efficiency from 89.06% to 96.21%, with maltodextrin and gum Arabic combination being highly efficient.

Tumbas Šaponjac et al. [26], used response surface methodology in order to obtain the highest encapsulation efficiency for polyphenol extracted from beetroot pomace in soy protein. These authors reported different values ranging from 60.26% to 85.03% as a function of wall-core ratio, extract dilution and mixing time.

Regarding the protein-phenolic interactions, it has been suggested that various type of molecular interactions may be involved, such as hydrophobic, electrostatic, van der Waals, and hydrogen bonding [27], depending on the protein and the phenolic type. As for sweet cherries extract, the major anthocyanin was found to be cyanidin 3-rutinoside [22], therefore the interaction site between the predominant whey protein and cyanidin 3-rutinoside seems to be located between strand D, EF loop and CD loop, as explained by Oancea et al. [16]. These authors identified the main amino acids from the binding sites of the protein directly facing the ligand: Leu^58^, Leu^87^, Asn^90^, and Met^107^.

### 3.3. Structure and Morphology of the Microencapsulated Powder

**Figure 1:**
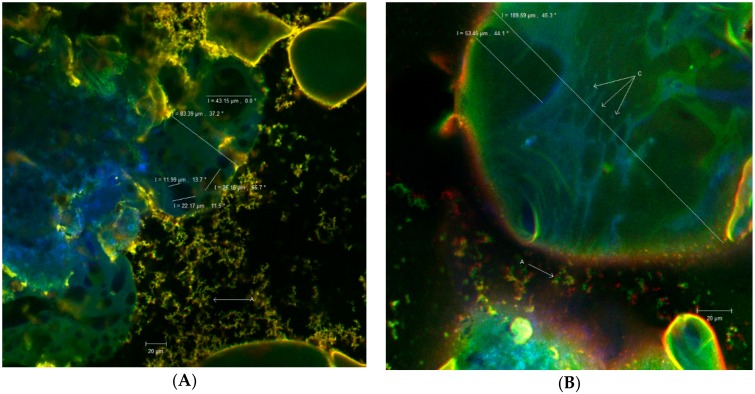
(**A**,**B**) Confocal microscopy images highlighting the double coacervation of anthocyanins from sweet cherries skins: A—Aggregates, C—Coacervates (The images taken over by the ZEN Black 2012 software).

Cherries are drupe type fruits containing anthocyanin pigments found in the epicarp and mesocarp. The strong absorption of anthocyanins in the visible field was determined between 465 nm and 550 nm [28] with an in vivo peak between 537 nm and 542 nm. The matrix formed between the skins of cherries anthocyanins and whey proteins, even in its native form, showed autofluorescence. The images taken over by the ZEN Black 2012 software revealed coacervates (marked with C) of spherical or elypsoidal shape, with various diameters (11.99 μm, 22.17 μm, 25.15 μm, 43.15 μm, 53.45 μm) (Figure 1).

These coacervates aggregate into large spherosomes (ranging from 83.39 μm to 189.59 μm). Thus, the anthocyanins shown in blue-green were subjected to a double encapsulation in the whey protein matrix, shown in yellow (Figure 1). However, from Figure 1 it is also possible to observe free, unencapsulated clusters (in green) marked with A, which can be correlated with the encapsulation efficiency.

### 3.4. Scanning Electron Microscopy

**Figure 2:**
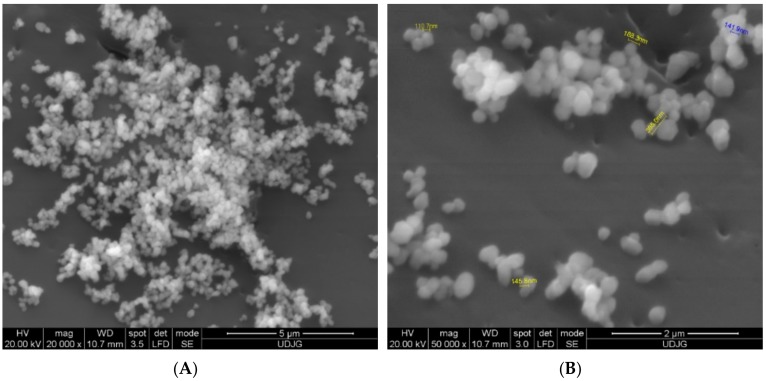
The SEM images of the microencapsulated anthocyanins from sweet cherries skins. (**A**) Direct view of aggregates; (**B**) Measurement of aggregates dimensions. (A—magnification ×20,000, B—magnification ×50,000)

The attempt of measuring nano-level dimensions of various aggregates is difficult especially when these are organic complexes due their sensitivity. As it is well known it is difficult to measure such dimensions by dispersing the aggregates in liquids. The main idea is to fix somehow the particles and to analyze them by means of scanning electron microscopy (SEM). For this study, a Qanta device had been used in order to measure the dimensions of microencapsulated anthocyanins. The best results with excellent view over the aggregates had been obtained by directly placing the organic powder on the bi-adhesive band that is used to fix the samples inside the analysis machine. As the bi-adhesive band is highly conductive the small aggregates are well defined in images offering an excellent view over their forms and dimensions (Figure 2A,B), as well as confirming their nano-level dimensions. As it can be seen from Figure 2A,B, the microcapsules were smooth, with different sizes, varying from 110.7 nm to 365 nm, showing some agglomeration and no dents on the surface, were well distributed, confirming that WPI-chitosan combination is an effective encapsulation material. Osorio et al. [29], have highlighted the importance of the smooth character of spheres obtained by micro-encapsulation, both for the stability of the encapsulated material and for its controlled release.

Pereira et al. [30] used a combination of methods to produce nanoparticles containing poly (D, L-lactic-co-glycolic) acid (PLGA) and phenolic extract of guabiroba. The nanoparticles were synthesized by combining the emulsion-evaporation and free-drying methods. The resultant nanoparticles presented an average particle diameter of 243.8 ± 79.7 nm. Also, Silva et al. [31], reported particle size values for PLGA 65:35 and 50:50 nanoparticles loaded with phenolic compounds and extracts to be between 140 and 250 nm.

### 3.5. Phytochemical Characterization of Microencapsulated Powder

The powder had a significant content in TPC, TFC and TAC of 267.03 ± 12.20 mg GAE/100 g D.W., 93.59 ± 4.55 mg CE/100 g D.W. and 14.48 ± 1.17 mg C3G/100 g D.W., respectively. The antioxidant activity was 85.37 ± 1.18 µmol Trolox/100 g D.W. Oancea et al. [16], reported a significant higher value for TAC of 31.95 ± 0.65 mg C3G/100 g D.W., but significant lower for TPC and TFC of 5.82 ± 0.26 mg GAE/100 g D.W. and 3.58 ± 0.73 mg CE/100 g D.W., respectively, for phytochemicals from sour cherries skins microencapsulated in WPI and acacia gum by coacervation and freeze-drying technique. Due to the higher content in TAC, these authors obtained a powder with a significant higher antioxidant activity of 480.58 ± 1.84 µmol TE/mg D.W. These authors used two combined encapsulation techniques, namely coacervation and freeze-drying, which allowed the encapsulation of a larger number of anthocyanins, but significantly lower number of polyphenols and flavonoids. Probably, the key factor that allowed encapsulation of a higher amounts of anthocyanins was the pH used in the coacervation step, which led to a greater stability of the selected compounds.

Kar et al. [32], used response surface methodology to optimize the encapsulation of anthocyanin pigment from black carrot juice in a core material formed by whey protein isolate (WPI), jackfruit seed starch (JSS) and an emulsifier similar to gum Arabic (NBRE-15). These authors suggested that the optimized condition for the selected variable were 27.8 Brix, 1:5 (WPI:JSS) and 0.3% (NBRE-15), yielding a powder with a significant content of anthocyanins (2766.61 mg/100 g D.W.) and antioxidant activity (290.56 µmol Trolox/g D.W.). These authors reported a similar encapsulation efficiency of 77.12%.

### 3.6. The Influence of Microencapsulated Anthocyanins on the Growth of L. casei 431^®^

During 21 days of storage, a decrease in the number of *L. casei* 431^®^ viable cells of all samples was observed (Table 1).

After 14 days of storage, no significant differences were found between samples B and C, but both samples were significantly different when compared with control (A). However, the viability of *L. casei* 431^®^ was significantly different in all the tested variants (*p* < 0.001) after 21 days of storage at 4 °C. If compared with time 0, from Table 1, it can be observed that the number of *L. casei* 431^®^ in the control sample decreased with approximatively 54% compared to the enriched sample B, where the reduction was 70%. An inhibitory effect of the powder was observed in sample C (10% addition) when compared to control (A) and B samples. Our results are in contrast with those reported by Oancea et al. [16], who suggested that microencapsulated anthocyanins from sour cherries skins extract had a stimulatory effect on the growth of *L. casei* 431^®^, leading to a preserving effect on the cell viability up to 10^10^ CFU·g^−1^ after 21 days of storage.

Food polyphenols are able to selectively modify the growth of susceptible microorganisms [33]. For example, Tabasco et al. [33] have noticed that *Lactobacillus plantarum* IFPL935 is capable of initiating the catabolism of flavan-3-ols. Smith et al. [34] explained the mechanisms by which bacteria can overcome inhibition by proanthocyanins, including modification/degradation, dissociation of polyphenol substrate complexes, polyphenol inactivation by high-affinity binders, as well as membrane modification/repair and metal ion sequestration. However, it must be kept in mind that although resistance to polyphenols is the first step bacteria have to go through to metabolize these compounds, resistance does not guarantee metabolic activity, as explained by Tabasco et al. [33].

It is well known that in order to confer health benefit, probiotic bacteria must arrive in intestine alive and in sufficient numbers, i.e., 6–7 log CFU/g of product [35]. Given the data presented in Table 1, it can be concluded that the obtained value-added yoghurt samples may have a probiotic effect due to the number of probiotic bacteria after 21 days of storage of at least 6 log CFU/g of product at concentration ranging from 5 to maximum 10%.

### 3.7. The Stability of Phytochemicals in Value-Added Food Products during Storage Test

The two value-added food products were tested for the stability of biologically active compounds and antioxidant activity during a storage test. Therefore, the yoghurt and marshmallows samples were tested for TAC and antioxidant activity during storage at 4 °C and room temperature, respectively.

**Figure 3:**
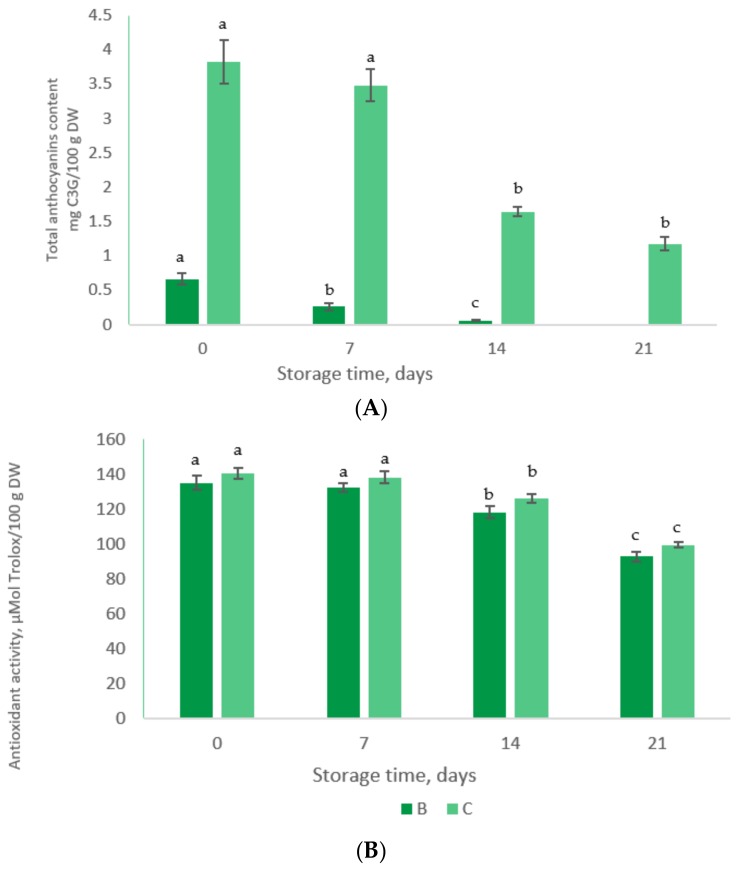
The stability of anthocyanins (**A**) and antioxidant activity (**B**) in yoghurt samples during storage test. Superscripts that for the same sample do not share the same letter (a, b, c) are statistically significant at *p* < 0.001.

In Figure 3 are given the TAC content and antioxidant activity in yoghurt samples during storage. It can be observed from Figure 3A that no anthocyanins were detected in samples B after 21 days. Similar results were reported by Robert et al. [36], who suggested that the microencapsulated anthocyanins disappear in yoghurt before seven days. Also, Coisson et al. [37] observed that during the addition of açai (*Euterpe oleracea*) juice to yogurt (10% *w*/*w*), the anthocyanins were stable for only two days at 4 °C.

Antioxidant activity decreases significantly (*p* < 0.001) in both yoghurt samples after 21 days of storage with ~32 % and ~29%, respectively (Figure 3B).

**Figure 4:**
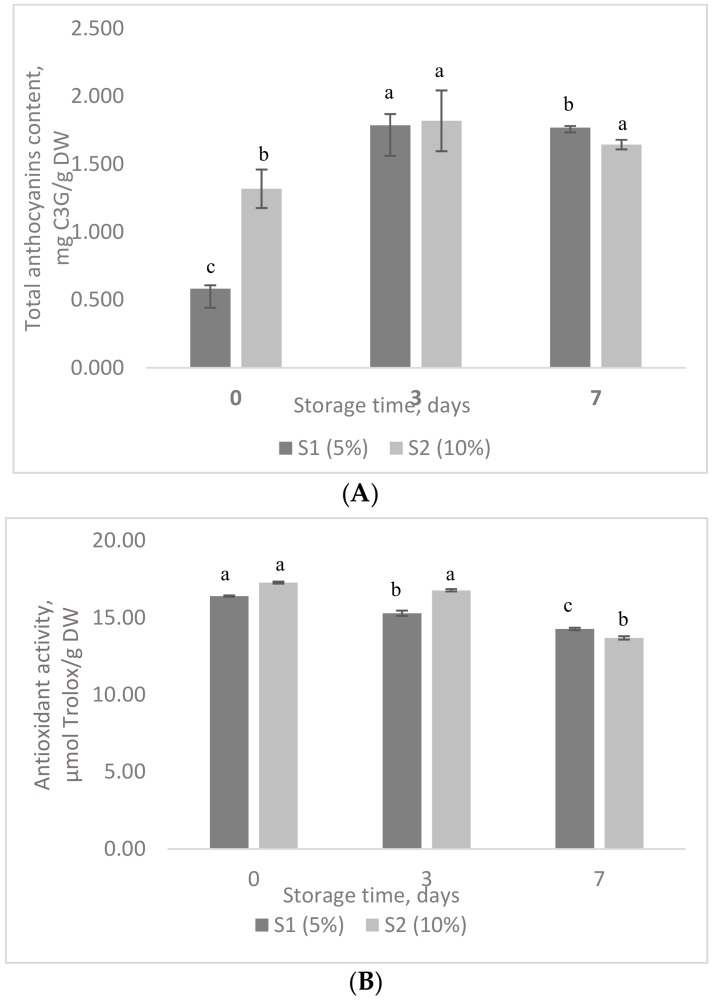
The stability of anthocyanins (**A**) and antioxidant activity (**B**) in marshmallows samples during storage test. Superscripts that for the same sample do not share the same letter (a, b, c) are statistically significant at *p* < 0.001.

Figure 4 shows the TAC and antioxidant activities of marshmallow samples during the storage test. A three-fold increase in TAC can be observed after three and seven days, suggesting their release from microcapsules (Figure 4A). Although TAC increased during storage (*p* < 0.001), antioxidant activity showed a slight variation, with a decrease of about 7% in the S1 and approx. 3% in S2 after 3 days of storage. After seven days, the antioxidant activity decreased with approx. 13% in S1 and 21% in S2 (Figure 4B). It can be appreciated that the behavior of microencapsulated anthocyanins depends highly on the matrix into which they are added.

The obtained results suggest that both products maintained the added value during storage, in terms of antioxidant activity. Therefore, our results are promising for the development of functional food products and supplements.

## 4. Conclusions

Considering the limited number of studies concerning sweet cherries by-products reuse, our study brings new opportunities to add value to different food products by valorization of skins through extraction and microencapsulation of anthocyanins. The extract showed a satisfactory phytochemical content in terms of polyphenolics and antioxidant activity, being further encapsulated in a whey protein isolate-chitosan matrix by freeze-drying. The anthocyanins were successfully encapsulated, with an efficiency of approximatively 78%. The microparticles showed variable dimensions, ranging from 12 μm to 54 μm, which aggregated into large spherosomes up to 190 μm. The microcapsules were smooth, with no dents on the surface, confirming that the whey protein isolate-chitosan matrix combination is an effective encapsulation material.

The functional properties of the microencapsulated powder were tested by adding as ingredient in two food products. Therefore, the viability of *L. casei* 431^®^ was tested during a storage period when an inhibitory effect was observed, especially at higher concentrations, up to 10%. However, these products showed satisfactory phytochemical and antioxidant activity during storage and may be considered as valuable candidates for the development of added-value food products.

## Figures and Tables

**Table 1:** Counts of *Lactobacillus casei* 431^®^ (log CFU·g^−1^) during 21 days of storage.

Storage Days	A	B	C
0	9.41 ± 0.32 ^aA^	8.07 ± 0.11 ^aB^	6.59 ± 0.15 ^aB^
7	9.34 ± 0.24 ^aA^	8.04 ± 0.14 ^bB^	6.23 ± 0.11 ^bB^
14	9.19 ± 0.14 ^bA^	7.64 ± 0.17 ^aB^	6.17 ± 0.13 ^bB^
21	9.07 ± 0.11 ^bA^	7.55 ± 0.15 ^bB^	6.14 ± 0.21 ^bC^

Values that on the same column do not share the same letter (a, b, c) are statistically different at *p* < 0.001 based on Tukey method and 95% confidence; Values that on the same row do not share the same letter (A, B, C) are statistically different at *p* < 0.001 based on Tukey method and 95% confidence; A—without addition (blank sample); B—with 5% microencapsulated anthocyanins from sweet cherry skins, C—with 10% microencapsulated anthocyanins from sweet cherry skins.
